# Multi-domain destructuring in the early phases of psychosis: a multicentric phenomenological and psychometric case-control study

**DOI:** 10.3389/fnbeh.2025.1614730

**Published:** 2025-07-22

**Authors:** Ottone Baccaredda Boy, Giuseppe Pierpaolo Merola, Andrea Patti, Bernardo Bozza, Dario Flaccomio, Marco Faldi, Giulia Pitt, Luca Papini, Vincenzo Pecoraro, Ilaria Noschese, Elisa Di Matteo, Dario Brugnolo, Camilla Ricci, Andrea Ballerini, Francesco Mauceri, Simone Tavano, Giulio Peroni, Sara Ciabattini, Sara Gori, Tiziana Pisano, Francesco De Cesaris, David Cohen, Valdo Ricca

**Affiliations:** ^1^Azienda Ospedaliera Universitaria Careggi Firenze, Florence, Italy; ^2^Hospital of Prato, Prato, Italy; ^3^Eating Disorder Clinic “Residenza Gruber”, Bologna, Italy; ^4^Santagostino Medical Center, Bologna, Italy; ^5^Child and Adolescent Psychiatric Unit, Neuroscience and Human Genetics Department, Meyer Children’s Hospital IRCCS, Florence, Italy; ^6^Azienda Ospedaliero-Universitaria IRCCS Bologna, Bologna, Italy; ^7^Child and Adolescent Psychiatry Unit, Institute IDEAL, APHP, Sorbonne University, Hôpital Pitié-Salpêtrière, Paris, France

**Keywords:** salience, psychosis, FEP, anxiety, depression, cannabis

## Abstract

**Introduction:**

The study aims to evaluate symptomatic differences through psychometric tools comparing patients in the early stages of psychotic development with those exhibiting a more established symptomatology. Our hypothesis was that the early phase in adolescent patients is accompanied by quantitatively and qualitatively distinct symptomatology compared to adults.

**Methods:**

We assessed 116 participants–consisting of 14 to 65 years old patients with psychotic or mood symptoms–using psychometric tools and a clinical interview. The tools explored psychotic, depressive and anxiety dimensions, to provide a multifaceted assessment of the recruited individuals and help at categorizing them into diagnostic subclasses.

**Results:**

We compared patients with psychotic symptoms (early-onset and lifetime) to patients with mood disorders (unipolar depression or bipolar disorder without psychotic symptoms). Psychotic symptoms intensity was significantly higher in the early-onset group compared to the lifetime group and was markedly greater than in the two other groups. It was also observed that the intensity of anxiety and depressive symptoms in the psychosis group were significantly higher in the early-onset subgroup.

**Conclusion:**

Our findings suggest that the clinical presentation of early-onset patients, typically striking in its symptomatology, is reflected by elevated scores on scales not routinely used for psychotic symptoms. This may be attributed to the pervasive destructuring of personality and reality characteristic of early psychotic experiences.

## 1 Introduction

Psychotic disorders are among the most disabling mental health conditions, accounting for 1.1% of total years of life lost due to disability (DALY), which brings significant costs to both families and mental health services (Lewine and Hart, 2020). The severe impact of psychosis is not only due to the nature of the disorder itself but also its early onset. Psychosis typically manifests between the ages of 18 and 25 in men, and between 20 and 28 in women (Kirkbride et al., 2017), with prodromal symptoms often emerging even earlier. The onset of psychosis during these years can be attributed to a combination of neurobiological and environmental factors, though many of the underlying causes remain poorly understood (Malone et al., 2010). Various models rooted in the diathesis-stress framework (Zuckerman, 1999) have sought to identify factors that predict the development of psychosis, aiming to create effective preventive tools (Parnas et al., 2005; Schultze-Lutter, 2009). The most established framework for psychosis development includes Ultra High Risk (UHR) and First Episode Psychosis (FEP) categories (Yung and McGorry, 1996; Yung and Nelson, 2013). Despite significant advancements in predicting psychosis, key aspects of its etiology remain unclear.

Recognizing early symptoms is critical for reducing the Duration of Untreated Psychosis (DUP), a strong predictor of poor outcomes (Howes et al., 2021). Psychosis onset is often preceded by a non-specific prodromal phase, which may involve emotional and cognitive disturbances, social withdrawal, and functional decline (McCutcheon et al., 2023; Yassin et al., 2024); these manifestations, apparently more subtle, cause a significant degree of impairment in individuals which gain awareness of them, leading to significant distress (Olvet et al., 2015). Individuals affected by schizophrenia spectrum disorders (SSD) are more likely to experience depressive and anxious symptoms compared to their peers, which can negatively impact their recovery potential (Catalán Alcántara et al., 2021; Tor et al., 2018).

Psychotic symptoms often co-occur with disturbances from various psychopathological areas. While positive symptoms are most commonly associated with psychosis, the disorder manifests in a range of cognitive and emotional disturbances (Addington et al., 2017). From a phenomenological perspective, these experiences align with the concept of “subapophanic” psychosis (Blankenburg, 2012), which lacks the revelatory aspect of delusions and emphasizes the loss of the natural self-evidence of the world. Schizophrenic individuals often struggle to construct reality and must work to re-establish basic experiential structures (Fusar-Poli et al., 2022). This process can lead to a range of symptoms, including cognitive, anxiety, and depressive disturbances, which might result in atypical psychoses with entirely new disease patterns, which in turn led to a rethinking of classification systems, describing these disorders as part of a spectrum (Fusar-Poli et al., 2014). Neurobiological and neuroanatomical variations further confirm this diversity (Godini et al., 2015; Huber and Gross, 1989; Koutsouleris et al., 2012). Recent critiques argue that the transition-to-psychosis model overemphasizes positive symptoms (van Os and Guloksuz, 2017), as many individuals show a gradual shift in symptomatology. While it is true that FEP patients display higher positive symptoms when compared to patients in more advanced states of psychosis (Del Bello et al., 2016), they also show evident anxious and depressive symptomatology (Coentre et al., 2017; McGinty and Upthegrove, 2020), as well as profound negative symptoms (Salazar de Pablo et al., 2023).

A critical feature of psychosis is the co-occurrence of symptoms with distinct biological mechanisms, such as the link between reduced dopamine in the prefrontal cortex and negative symptoms (Wu et al., 2024). Recent studies suggest that anxiety and depression might be primary sources of distress, potentially contributing to apophanic experiences (Fusar-Poli et al., 2022). These findings suggest that these symptoms may not be solely prodromal or early indicators of a specific disorder, but could also mark the onset of different psychopathological pathways (Spooner et al., 2020).

Therefore, it is crucial to identify which factors best predict the progression toward psychosis rather than other mental disorders. Aberrant salience (AS) refers to the dysfunctional assignment of significance to neutral or irrelevant stimuli, driven by dopaminergic dysregulation (Kapur, 2003; Kapur et al., 2005). This mechanism may contribute to the development of psychotic symptoms, such as delusions or hallucinations, by making trivial cues appear overly meaningful. While aberrant salience is a well-established concept, its clinical application remains limited, likely due to uncertainties about its specificity (Patti et al., 2024) and its unclear relationship with disease phase and diagnosis, showing high values in both UHR and FEP patients (Pelizza et al., 2021), as well as in both SSD and non-SSD patients (Spark et al., 2021). However, these limitations should not undermine its potential as an early detection tool.

The notion that AS may serve as an early indicator of a cognitive destabilization potentially leading to psychosis enhances the conceptual frameworks from which it originates. Adolescence appears to be the most relevant period to test this hypothesis, as it is during this phase that these phenomena are most likely to emerge, and adolescents are in fact known to have higher levels of AS (Lisi et al., 2021). Importantly, in young people, the symptoms of mental disorders often resist clear diagnostic classification, instead manifesting as a complex and overlapping constellation of clinical features (Scott et al., 2012). In early stages, distinguishing between psychotic and mood disorders provides limited practical value, especially given the high degree of symptom co-occurrence; recent models–such as the transdiagnostic psychosis phenotype (van Os and Reininghaus, 2016)–suggest that even symptoms that were previously characterized as pathognomonic do not yield definitive results in predicting disease trajectory (McGorry et al., 2018; Spooner et al., 2020). The concept of developmental heterotopy emphasizes that symptoms should be viewed not in isolation but as interrelated elements within a broader complex; overlooking some of these features may critically undermine both the identification and staging of these disorders (Hartmann et al., 2021; Scott et al., 2024).

### 1.1 Aims

The aim of this study is therefore to evaluate the symptomatic differences between patients in the early stages of psychotic development and a group with already structured symptomatology, using some of the most commonly employed psychometric scales in clinical practice and research, thus evaluating both psychosis dimensions and adjacent psychopathological constructs as well.

## 2 Materials and methods

Patients were interviewed at the mental health units and neurology outpatient clinics of the Meyer Children’s Hospital in Florence and the Pitié-Salpêtrière Hospital in Paris, at the outpatient clinics and Day Hospital services of the Careggi University Hospital in Florence. Additionally, part of the sample was recruited from public hospitals in the Tuscany Central Area, which had been involved in earlier phases of the study.

The sample was selected during outpatient visits and hospitalizations over a four-year period. All participants underwent a clinical and sociodemographic interview, along with a series of psychometric tests aimed at dimensionally assessing psychotic and general symptomatology.

Patients were stratified based on age (“M” for adolescents aged 14–18 years; “A” for adults aged over 18) and a broad diagnostic categorization. The first major group consists of adult patients (coded as A), further divided into two distinct subgroups based on diagnosis: either psychosis spectrum disorders (A.PSI) or mood disorders in absence of overt psychotic features (A.MOOD).

The same subgrouping method was applied to adolescent patients (coded as M, aged 14–18), resulting in two analogous subgroups: M.PSI and M.MOOD. This categorization resulted in four clearly defined study groups: A.PSI, A.MOOD, M.PSI, and M.MOOD.

All diagnoses were made by physicians and had a primarily clinical purpose. Diagnoses were made according to DSM-5 criteria (American Psychiatric Association, 2013), using all relevant disorders included in the schizophrenia spectrum and other psychotic disorders groups. Recruited patients were administered a comprehensive set of questionnaires covering various domains of both general and specific psychopathology. All included scales had been previously validated for both adult and adolescent cohorts (14 years and older) (Núñez et al., 2021; Pelizza et al., 2018; Rodríguez-Testal et al., 2022). When selecting the questionnaires, differences between the Italian and French versions were taken into account; where relevant, scores were adjusted accordingly during the scoring phase.

Two sets of scales were employed: instruments investigating the psychotic dimension–Positive and Negative Syndrome Scale (PANSS) (Kay et al., 1987), Community Assessment of Psychic Experiences (CAPE) (Daneluzzo et al., 2008), Arbeitsgemeinschaft für Methodik und Dokumentation in der Psychiatrie (AMDP) system (Guy and Ban, 1982; Stieglitz et al., 2017) and aberrant salience inventory (ASI) (Cicero et al., 2010) and scales centered around other forms of psychopathology–Montgomery-Åsberg Depression Rating Scale (MADRS) (Montgomery and Åsberg, 1979), Young Mania Rating Scale or YMRS (Conti, 2002; Favre et al., 2003; Young et al., 1978) and Hamilton Anxiety Rating Scale (HAM-A) (Hamilton, 1959).

Finally, a shorter version of the Cannabis Experience Questionnaire, which has already been widely employed in substance use evaluation in patients with psychotic symptoms (Birnbaum et al., 2019), was used to assess lifetime cannabis exposure.

Together, these tools provided a comprehensive assessment of psychotic, depressive, and anxiety-related symptoms, allowing for a detailed characterization of the participants’ psychopathological profiles. All questionnaires were administered by the same physicians responsible for the clinical management of the patients. This ensured an accurate assessment and a precise score, also turning the test into an opportunity to evaluate the patient from another perspective and guide the clinical decision-making process.

A table with a glossary for the employed psychometric scales and their subdimensions is reported in Supplementary Table 1.

### 2.1 Data analysis

The statistical software RStudio was used for the analyses (Allaire, 2012). The mean and standard deviation (SD) were calculated for continuous variables, while frequency and percentage were used for categorical variables. All comparisons were conducted across the four diagnostic-age groups (A.PSI, A.MOOD, M.PSI, M.MOOD). The difference between groups was calculated using Chi-square test for categorical variables, with standardized residuals analysis used to identify the outliers (with values > 2/> −2 underscoring a strong deviation); Kruskal–Wallis and Fisher tests were used for continuous variables comparison.

Shapiro–Walk tests were used to evaluate the normality of distribution. Kruskal–Wallis test was then employed to assess differences between groups, which were furtherly investigated by conducting Dunn’s *post-hoc* test with Bonferroni correction. Two linear regression analyses were conducted to investigate whether psychotic symptom severity (measured by total PANSS score, TOTPANSS) predicts anxiety (HAMATOT) and depressive symptoms (MADRSTOT), and whether these associations are moderated by age; TOTPANSS served as independent variable, while HAMATOT and MADRSTOT served as the dependent ones. The rationale for this lies in the hypothesis that symptoms from these three areas might be proportionally represented, especially in adolescents. Additionally, mediation analysis was performed to investigate the indirect effects of the studied variables and their potential pathways of direct and indirect influence. Inferences drawn upon mediation analyses on cross-sectional data have inherent limitations, but in certain instances it is justified to utilize this method (O’Laughlin et al., 2018).

Reliability analysis was conducted using Cronbach’s alpha coefficient (Cronbach’s alpha). A probability level of *p* < 0.05 was selected to determine statistical significance.

## 3 Results

A total of 116 participants were recruited, 56 in the adolescent subgroup and 60 in the adult one. Of the 56 patients under 18 years old, 23 were recruited in the Neuropsychiatric Units from Pitié-Salpêtrière Hospital, while the remaining ones were recruited at Meyer’s Children Hospital. For the adult group, 37 were recruited at Careggi Hospital while the remaining 23 (38.3%) were recruited in Toscana Centro Hospitals. The sociodemographic and anamnestic characteristics of the sample are listed in Table 1. Group comparisons were systematically performed across all four subgroups.

**TABLE 1:** Sociodemographic variables.

	M.PSI (*n* = 28)	M.MOOD (*n* = 28)	A.PSI (*n* = 30)	A.MOOD (*n* = 30)	TOT (*n* = 116)
***n* (%)**					
Male sex	11 (0.39)	4 (0.14)	15 (0.5)	12 (0.4)	43 (0.37)
Cannabis	11 (0.39)	7 (0.25)	10 (0.33)	11 (0.37)	39 (0.34)
Family history	15 (0.54)	17 (0.61)	13 (0.47)	10 (0.33)	56 (0.48)
Antidepressants	8 (0.29)	11 (0.39)	11 (0.37)	21 (0.7)	52 (0.45)
Mood stabilizers	10 (0.36)	13 (0.46)	12 (0.4)	12 (0.33)	47 (0.39)
Antipsychotics	23 (0.82)	15 (0.54)	23 (0.83)	12 (0.4)	75 (0.65)
Anxiolytics	13 (0.46)	12 (0.43)	14 (0.47)	14 (0.47)	53 (0.46)
Bipolar disorder	10 (0.36)	5 (0.18)	11 (0.37)	8 (0.27)	44 (0.38)
Outpatient	6 (0.21)	11 (0.39)	9 (0.3)	7 (0.23)	33 (0.28)
**Mean of years (SD)**					
Education	9.5 (1.58)	10.14 (1.15)	12.17 (3.41)	12.27 (3.39)	11.06 (2.87)
Age	15.61 (1.31)	15.64 (1.16)	34.83 (14.11)	41.03 (16.63)	27.16 (15.84)
Hospitalizations	1.61 (1.66)	1.29 (1.54)	3.31 (6.77)	1.45 (2.98)	1.92 (3.93)
Age of onset	10.5 (4.57)	11.36 (3.87)	23.1 (8.45)	23.37 (10.3)	17.29 (9.56)

Cannabis stands for “Cannabis exposure,” as assessed through item 4a of CEQ. Family history stands for “Family history of psychiatric disease.” M.PSI, minors with psychotic symptoms; M.MOOD, minors with mood symptoms; A.PSI, adults with psychotic symptoms; A.MOOD, adults with mood symptoms; TOT, total sample.

Chi-square test showed a significant imbalance in the male-female ratio across groups (*p* = 0.02), but standardized residuals did not detect a strong deviation. Significant differences were detected also in terms of antidepressant (*p* = 0.01) and antipsychotic (*p* < 0.001) treatment. According to a follow-up standardized residual analysis, for what concerned antidepressant treatment Group A.MOOD was overrepresented (residual = 2.14), while in terms of antipsychotic treatment, A.PSI and M.PSI were less represented than expected in the “Antipsychotic-free” category. The groups appeared to be homogeneous in terms of recruitment setting: no significant difference was detected when comparing the outpatient vs. hospitalized proportion across each group (*p* = 0.44, see Table 1).

Kruskal–Wallis tests revealed a significant difference in terms of Education, Age and Age of onset (*p* < 0.001). No significant difference was found in terms of Hospitalizations (*p* = 0.2). Fisher’s test did not reveal a significant difference in terms of cannabis exposure (*p* = 0.71).

Table 2 reports the average scores at the employed psychometric scales.

**TABLE 2:** Scores at the general psychopathology scales were compared through Kruskal–Wallis test to investigate differences among groups.

	M.PSI (*n* = 28)	M.MOOD (*n* = 28)	A.PSI (*n* = 30)	A.MOOD (*n* = 30)	TOT (*n* = 116)	Kruskal–Wallis test
**Mean (SD)**						***p*-value**
ASI_EIE	8.89 (4.9)	9.64 (3.58)	5.03 (3.44)	3.1 (3.3)	6.58 (4.66)	0.001**
ASI_UE	3.36 (2.42)	2.93 (2.55)	2.87 (1.68)	2.47 (1.66)	2.9 (2.1)	0.54
ASI_SS	3.11 (2.23)	3.04 (1.91)	2.87 (1.68)	1.87 (1.43)	2.71 (1.87)	0.03*
ASI_TOT	15.11 (7.98)	14.89 (6.62)	15.8 (6.64)	12.6 (6.54)	14.59 (6.97)	0.31
MADRSTOT	21.64 (8.33)	20.96 (10.7)	13.53 (9.39)	14.23 (11.18)	17.7 (10.44)	0.57
MRSTOT	7.25 (5.49)	4.11 (3.37)	7.43 (6.3)	4.91 (7.47)	6.01 (5.86)	0.03*
HAMATOT	13.25 (6.48)	11.39 (7.21)	8.97 (6.66)	9.87 (7.3)	10.88 (7.1)	< 0.01**
pPANSSTOT	16.18 (7.6)	7.43 (1.2)	11.7 (5.18)	9.04 (6.97)	11.13 (6.58)	< 0.01**
nPANSSTOT	22.64 (9.84)	10.82 (4.95)	12.77 (6.36)	11.16 (4.6)	14.41 (8.29)	< 0.01**
gPANSSTOT	43.32 (14.42)	29.75 (8.23)	31.3 (10.42)	26.74 (8.62)	33.03 (12.36)	< 0.001***
TOTPANSS	82.14 (24.32)	48.1 (12.1)	55.87 (17.33)	47.57 (14.95)	58.84 (22.6)	< 0.001***

ASI, aberrant salience inventory; ASI_EIE, ASI–enhanced interpretation and emotionality; ASI_UE, ASI–unveiling experiences; ASI_SS, aberrant salience inventory–senses sharpening; ASI_TOT, ASI–total score; MADRSTOT, Montgomery-Åsberg Depression Rating Scale total score; MRSTOT, Mania Rating Scale total score; HAMATOT, Hamilton Anxiety Rating Scale total score; pPANSS, PANSS positive subscale; nPANSS, PANSS negative subscale; gPANSS, PANSS general subscale; TOTPANSS, PANSS total score. ***p < 0.001, **p < 0.01, *p < 0.05.

**FIGURE 1:**
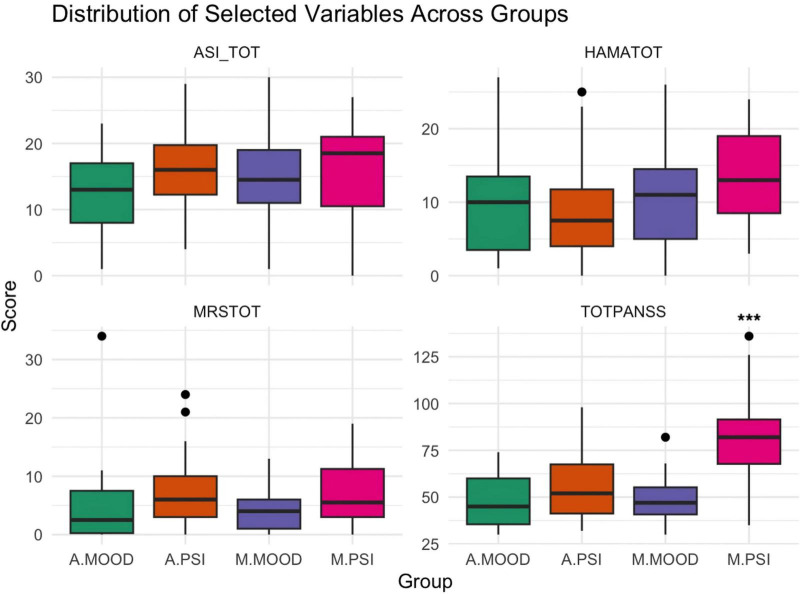
Boxplots from the Krusal-Wallis analysis results are displayed, showing the asymmetrical distribution of data for some of the studied variables. ***p-value < 0.001 as calculated through Dunn post-hoc analysis comparing M.PSI group to the other three.

A Shapiro–Wilk test for normality was performed on the continuous variables across the 4 groups, revealing that most variables deviated from normality, with significant *p*-values (*p* < 0.05) for all groups in several scales, including most PANSS scales (Figure 1).

Dunn’s *post-hoc* test with Bonferroni correction revealed notable pairwise differences in several comparisons. The A.MOOD group consistently showed lower scores on ASI and PANSS total scores compared to the others (*p* < 0.001). M.PSI group had the highest scores in Hamilton Anxiety Scale total score when compared to all other groups (*p* < 0.01). For what concerned PANSS subscales, the comparison of the positive subscale between A.MOOD and M.PSI (*Z* = −5.25, *p* < 0.0001) showed a strong difference, with A.MOOD significantly lower than M.PSI. A.MOOD consistently scored lower than the other groups also on negative (*p* < 0.001) and general PANSS subscales (*p* = 0.03 vs. A.PSI, *p* = 0.02 vs. M.PSI).

Other significant differences were found between A.PSI and M.PSI for ASI_EIE (*Z* = −3.84, *p* = 0.0001) and A.MOOD and M.PSI for the ASI subscale on unveiling experiences (*Z* = −1.70, *p* = 0.090). Krusal–Wallis analyses were also conducted for AMDP subscales, whose average scores are reported in Supplementary Table 2; the analyses revealed a significant difference across groups for all the studied variables (*p* < 0.05). M.PSI demonstrated statistically significant elevations (*p* < 0.01 to *p* < 0.0001) in psychopathological dimensions such as disorders of orientation (vs. A.MOOD: *p* = 0.01), perceptual disturbances (vs. A.PSI: *p* = 0.01), and delusions (vs. M.MOOD: *p* < 0.0001), with effect sizes ranging from 3.32 to 5.76. M.PSI showed the strongest contrasts, particularly in cognitive/perceptual disturbances (disturbances of attention/memory: *p* < 0.0001; disturbances of affectivity: *p* < 0.0001). A.PSI displayed intermediate results, showing significant differences from M.PSI but fewer contrasts with mood groups.

Only one minor significant difference was detected for what concerned CAPE scores (M.PSI had higher scores than A.MOOD group at the CAPEposF subscale, *p* = 0.01, the one for positive symptom frequency), while for all the other comparisons there was no significant difference.

For what concerns regression analyses, in the model predicting MADRSTOT the interaction between TOTPANSS and Age was statistically significant (*p* = 0.01), suggesting that age moderates the relationship between psychotic symptoms and depressive symptoms; on the other hand, HAMATOT model showed just a trend toward significance (*p* = 0.08), while not reaching conventional significance levels. Both models explained a modest proportion of variance (adjusted *R*^2^ = 0.21–0.23); the results are summarized in Supplementary Table 3.

**FIGURE 2:**
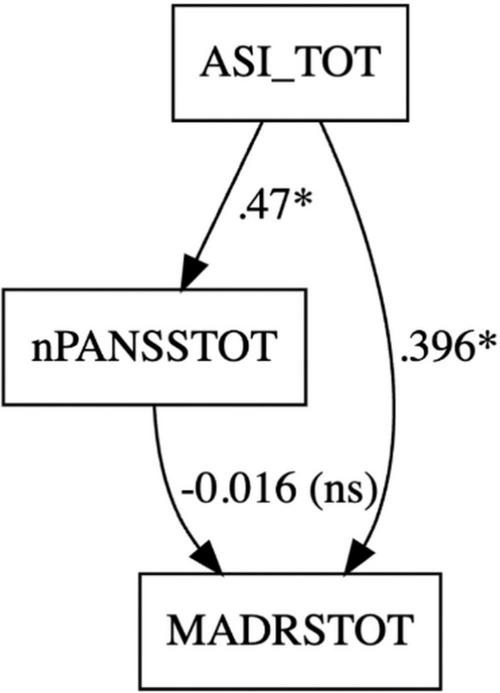
Mediation analysis with ASI_TOT (indipendent variable), MADRSTOT (dependent variable), and nPANSSTOT (mediator) was carried out to assess whether negative symptoms mediated the effect of aberrant salience scores on depressive symptoms scores. *p-value < 0.05.

Finally, mediation analysis results revealed that ASI total score has a significant direct effect on MADRS, but by placing the negative subscale of PANSS as a mediator, it does not significantly mediate this relationship (Figure 2). The Average Causal Mediation Effect (ACME), representing the indirect effect of ASI_TOT on MADRSTOT through nPANSSTOT, was estimated at −0.016 (95% CI: −0.1114 to 0.10, *p* = 0.738), indicating no significant mediation effect.

Conversely, the Average Direct Effect (ADE) of ASI on MADRS, excluding the mediator, was 0.3958 (95% CI: 0.1143 to 0.66, *p* = 0.008), demonstrating a significant positive direct relationship between ASI and MADRS.

The total effect of ASI on MADRS, which includes both direct and indirect effects, was estimated at 0.3798 (95% CI: 0.0816 to 0.66, *p* = 0.024), while the proportion of the total effect mediated by nPANSSTOT was −0.0422 (95% CI: −0.5821 to 0.31, *p* = 0.754), suggesting that the mediation pathway through nPANSSTOT does not account for a meaningful portion of the total effect.

## 4 Discussion

The presence of affective and anxious symptomatology in adolescent SSD patients, which constituted the main scope of the study, was confirmed; the severity of these symptoms was positively correlated with the intensity of psychotic symptoms (Hartley et al., 2013; Karpov et al., 2021). This supports the idea that a depressive matrix may be involved in the development of first-episode psychosis (Calderon-Mediavilla et al., 2021; Pelizza et al., 2023), especially when such symptoms arise and persist during adolescence. Nevertheless, prominent depressive symptoms appeared to be present also in older patients: regression analysis revealed that in adults with SSD age may influence the relationship between overall psychotic symptom burden and affective symptoms, particularly depression (manic symptoms, as measured through MRS, never reached a clinically significant threshold throughout the whole sample). Provided that the younger patients in our sample more closely fitted the criteria for FEP, this finding is not in line with recent studies: it was shown that more than 60% of children and adolescents with FEP exhibit negative symptoms, with a prevalence significantly higher than in adults with FEP (Salazar de Pablo et al., 2023).

Our data more accurately matches these observations when observing the differences found in the nPANSS scores between adolescents and adults. However, mediation analysis did not support the role of negative symptoms in mediating the relationship between AS and depression, confirming the direct effect of AS on depressive symptoms. The reason for this apparent discrepancy might reside in the different characteristics of affective episodes in these patients, as while we know that depressive symptoms in adolescents may reflect difficulties in adapting to their social environment, influenced by an anomalous world experience, this is not necessary true for adults; notably, adolescents with psychotic symptoms display a higher degree of suicidal thoughts (O’Hare et al., 2024). This could be linked to both positive symptoms like AS (Lisi et al., 2021; Poletti et al., 2022) and negative symptoms like perplexity (Vodušek et al., 2014) or the loss of self-evidence characteristic of subapophanic psychoses (Guardascione, 2023).

For what concerns anamnestic and clinical characteristics, they somewhat confirmed the preliminary group allocation based on clinical evaluation, showing that patients with mood disorders were more likely to be prescribed antidepressants, while those with psychotic disorders were rarely antipsychotic-free compared to mood disorder patients. Age-related variables significantly differed between adults and adolescents, except for the number of hospitalizations, which remained similar across age groups. This suggests that adolescents had a substantial number of hospitalizations despite their young age. The sample appeared to be homogeneous in terms of recruitment unit: the proportion of patients evaluated in Day Hospital or outpatient setting ranged from 21% to 39% of the subgroup; the remaining part of each subgroup was evaluated during an hospitalization. This prevents a possible bias regarding symptom intensity, as a higher severity of the clinical picture could be expected in the hospitalized patients, due to a state phase criterion.

The psychotic symptom scores were particularly interesting, as the groupings were based on clinical evaluations rather than psychometric scales. For instance, adolescents from the SSD subgroup showed significantly elevated positive and negative symptoms at the PANSS, while adults in the psychosis spectrum displayed relatively low scores, with only a minor difference compared to the mood groups. This discrepancy between adolescent and adult psychotic patients might be attributed to the different stages of the disease or to an optimized treatment regimen in adolescents (Hermes et al., 2012; Johnsen et al., 2010). Similar patterns were observed in other psychotic symptom scales, with the M.PSI group consistently scoring higher in AMDP, but the CAPE only revealed significant differences in the positive symptoms distress subdimension. The lack of elevation in other CAPE subscales could be due to the scale’s design, which is intended to measure psychosis-like experiences in the general population (Daneluzzo et al., 2008). Overall, the results suggested that patients in earlier disease phases exhibit more severe psychotic symptoms than those in later disease stages (Bunk et al., 1999; Del Bello et al., 2016).

The mean ASI values exceeded the cut-off in all groups except adults with mood disorders without psychotic features. However, there were no significant differences between the acute psychotic group and the mood disorders group, which may indicate that AS persists even in chronic stages of the disorder (Vaidya et al., 2024). These results support a conceptualization of AS more as a trait aspect (Godini et al., 2015) rather than a state-based feature (Nelson et al., 2019). Moreover, they support previous findings reporting comparable AS levels in both SSD and non-SSD FEP patients (Spark et al., 2021) and between adults with different psychiatric diagnoses (Ballerini et al., 2022), stemming from the transnosographic dimensional aspect of AS. The correlation between anxious and psychotic symptoms was even more pronounced, aligning with the view that anxiety symptoms develop in conjunction with psychotic symptoms (Achim et al., 2011; Lysaker and Salyers, 2007). AS is associated with more frequent positive psychotic-like experiences and elevated anxiety levels in both psychotic patients and healthy controls (Merola et al., 2024). The prevalence of comorbid depression and anxiety is as high as 17.7% in SSD individuals; anxiety comorbidity alone is more common, if compared to depression–37.4% vs. 19% (Zhao et al., 2024); in up to 41% of patients, also panic attacks are present (Veras et al., 2025). Rates are closer to our findings–in which we observed a slightly stronger correlation with affective disorders–if we consider FEP, with 29% of patients showing anxiety symptoms compared to 23% experiencing depressive ones (Wilson et al., 2020). The reality distortion seen in these patients is often described as a form of “anguish,” which can be mistaken for anxiety-like symptoms, while also leading to depressive ones. Classical authors such as Conrad and Jaspers formulated concepts like “Trema” and “Wahnstimmung” to describe this unease and fear before and during psychosis onset (Conrad, 1958; Jaspers, 1913). The simultaneous emergence of anxious and depressive symptoms can indicate a profound “de-structuring” of the self, leading to a break in the continuity of identity, which alters the lived experience of time in psychosis (Allé et al., 2016; Northoff et al., 2021; Pionke-Ubych et al., 2022).

To summarize, our sample clearly shows the multilayered impact of the psychosis onset, with prominently elevated psychopathological scores in several areas, not necessarily bound to the traditional psychosis spectrum; coexistence of aberrant salience, depressive and anxiety symptoms, already actively observed in early phases (Nastro et al., 2023), generates even more intricate clinical pictures in early phases of SSD. Such diversity gradually fades–as a curve that flattens and declines–once the disease progresses to chronicity: individuals with long-standing psychosis and stable treatment only occasionally presented with multilayered symptomatology.

## 5 Conclusion

This study highlights key differences between patients in early and advanced phases of psychosis, particularly regarding psychotic, depressive, and anxious symptoms. FEP patients tend to present more intense and variable affective symptoms, often tied to the emotional upheaval accompanying psychosis onset. In contrast, chronic patients display more stabilized anxious and depressive symptoms.

These findings underscore the heterogeneity of symptom expression in psychosis, particularly across affective, anxious, and perceptual domains. Younger patients showed more pronounced affective and subjective distress, while chronic cases exhibited elevated cognitive-perceptual and psychotic symptoms. These patterns highlight the importance of assessing symptom dimensions across stages of illness. Recognizing these differences may inform more tailored clinical assessments and improve our understanding of symptom evolution and treatment needs in psychotic disorders.

## Data Availability

The raw data supporting the conclusions of this article will be made available by the authors, without undue reservation.
