# Supplementary material for: Multi-domain destructuring in the early phases of psychosis: a multicentric phenomenological and psychometric case-control study
Source: Front Behav Neurosci. 2025 Jul 22;19:1614730. doi: 10.3389/fnbeh.2025.1614730 (PMC12321885; doi:10.3389/fnbeh.2025.1614730)
Supplement: Supplementary file 1 [file Data_Sheet_1.pdf]

Appendix

Scales and subscales	Psychopathological area
AMDPDistCosc	Disorders of consciousness
AMDPDistOrient	Disorders of orientation
AMDPDistAttent	Disorders of attention
AMDPFormDist	Formal thought disorders
AMDPFears	Fears and phobias
AMDPDelusions	Delusions
AMDPPerceptDis	Sensory-perception disorders
AMDPSelfDis	Self/ego disorders
AMDPAffectDis	Affectivity disorders
AMDPEnergyDis	Energy disorders
AMDPCircadianDis	Circadian disorders
AMDPOtherDis	Other disorders
ASI_EIE	Increase in interpretativeness and emotions
ASI_UE	Revelatory experiences/apophenia
ASI_SS	Refinement of senses
ASI_TOT	Total score
pPANSS	Positive symptoms
nPANSS	Negative symptoms
gPANSS	General symptoms
TOTPANSS	Total score
CAPEposF	Positive dimension (frequency)
CAPEposD	Positive dimension (distress)
CAPEdepF	Depressive dimension (frequency)
CAPEdepD	Depressive dimension (distress)
CAPEnegF	Negative dimension (frequency)

CAPEnegD	Negative dimension (distress)

Supplementary Table 1: glossary for the dimensions and subdimensions of the employed psychometric scales.

	M.PSI (n=28)	M.MOOD (n=28)	A.PSI (n=30)	A.MOOD (n=30)	TOT (n=116)
**AMDPDistOrient**	1,57 (3,26)	0,04 (0,19)	0,13 (0,57)	0,33 (1,65)	0,51 (1,91)
**AMDPDistAttent**	6,07 (4,66)	1,89 (2,79)	0,83 (1,97)	0,57 (1,5)	2,28 (3,65)
**AMDPFormDist**	7,04 (6,61)	1,54 (1,88)	1,33 (3,15)	0,83 (1,78)	2,63 (4,55)
**AMDPFears**	5,32 (5,21)	2,75 (5,48)	1,63 (2,03)	2,37 (3,91)	2,98 (4,5)
**AMDPDelusions**	6,54 (7,44)	0,07 (0,38)	2,7 (4,08)	1,43 (2,46)	2,77 (5,08)
**AMDPPerceptDis**	2,64 (3,28)	0,46 (1,53)	0,87 (2,22)	0,47 (2,37)	1,09 (2,56)
**AMDPSelfDis**	2,14 (2,38)	0,79 (1,37)	0,43 (1,45)	0,87 (3,88)	1,04 (2,55)
**AMDPAffectDis**	23,75 (11,89)	19,29 (12,13)	8,2 (9,52)	7,7 (11,57)	14,5 (13,16)
**AMDPEnergyDis**	6,21 (4,4)	3,54 (2,76)	1,63 (2,33)	1,73 (2,57)	3,22 (3,58)
**AMDPCircadianDis**	1,29 (1,44)	0,86 (1,21)	0,37 (0,89)	0,5 (0,94)	0,74 (1,17)
**AMDPOtherDis**	9,67 (4,17)	4,89 (4,32)	2,1 (2,99)	2,33 (3,98)	4,62 (4,88)
**CAPEposF**	1,95 (0,65)	1,73 (0,48)	1,82 (0,54)	1,54 (0,53)	1,76 (0,56)
**CAPEposD**	1,67 (0,67)	1,55 (0,47)	1,94 (0,75)	1,55 (0,6)	1,68 (0,65)
**CAPEdepF**	2,43 (0,78)	2,74 (0,76)	2,33 (0,68)	2,42 (0,74)	2,48 (0,75)
**CAPEdepD**	2,26 (0,83)	2,63 (0,74)	2,66 (0,85)	2,56 (0,75)	2,53 (0,8)
**CAPEnegF**	2,26 (0,7)	2,28 (0,62)	1,95 (0,53)	2,11 (0,69)	2,15 (0,64)
**CAPEnegD**	1,9 (0,69)	2,27 (0,76)	2,22 (0,81)	2,21 (0,77)	2,15 (0,76)

Supplementary Table 2. Psychometric scales evaluating psychotic domains through AMDP (Arbeitsgemeinschaft für Methodik und Dokumentation in der Psychiatrie) and CAPE (Community Assessment of Psychic Experiences) subdimensions.

Predictor	MADRSTOT (Estimate)	Std. Error	p-value	HAMATOT (Estimate)	Std. Error	p-value
**Intercept**	6.6388	2.5678	0.0111*	2.4105	1.6825	155
**TOTPANSS**	0.1896	0.0406	<0.001*	0.1448	0.0266	<0.001*
**R^2**	0.1719			0.2201		
**Residual SE**	9.514			6.234		
**F-statistic**	21.8		<0.001*	29.64		<0.001*

Supplementary Table 3. Linear regression results to assess the effect of PANSS on MADRS and Hamilton scores; *: p-value < 0.005.
